# Effect of Malnutrition on the Expression of Cytokines Involved in Th1 Cell Differentiation

**DOI:** 10.3390/nu5020579

**Published:** 2013-02-19

**Authors:** Cristina González-Torres, Haydeé González-Martínez, Angel Miliar, Oralia Nájera, Jaime Graniel, Verónica Firo, Catalina Alvarez, Edmundo Bonilla, Leonor Rodríguez

**Affiliations:** 1 Department of Health Sciences, Autonomus Metropolitan University-Iztapalapa, San Rafael Atlixco 186, CP 09340, México D.F., Mexico; E-Mails: haydeeglz77@yahoo.com.mx (H.G.-M.); mundo@xanum.uam.mx (E.B.); leor@xanum.uam.mx (L.R.); 2 Postgraduate Study Section, High School of Medicine, Instituto Politécnico Nacional, Plan de San Luis y Díaz Mirón s/n, CP 11340, México D.F., Mexico; E-Mail: miga67@prodigy.net.mx; 3 Department of Health Care, Autonomus Metropolitan University-Xochimilco, Calz. del Hueso 1100, CP 04960, México D.F., Mexico; E-Mail: onajera@correo.xoc.uam.mx; 4 Pediatric Hospital-Iztapalapa, Servicios de Salud Gobierno del Distrito Federal, Calzada Ermita Iztapalapa 780, CP 09070, México D.F., Mexico; E-Mail: granielgj@yahoo.com.mx; 5 General Hospital of Mexico, Servicio de Pediatría, Urgencias y Terapia Intensiva, Secretaría de Salud, Dr. Balmis 148, CP 06726, México D.F., Mexico; E-Mails: veronicarfr@hotmail.com (V.F.); acatalinaae_med@yahoo.com.mx (C.A.)

**Keywords:** malnutrition, Type 1 immunological response, cytokines

## Abstract

Malnutrition is a common cause of secondary immune deficiency and has been linked to an increased susceptibility to infection in humans. Malnutrition specifically affects T-cell-mediated immune responses. The aim of this study was to assess in lymphocytes from malnourished children the expression levels of IL-12, IL-18 and IL-21, molecules that induce the differentiation of T cells related to the immunological cellular response (Th1 response) and the production of cytokines related to the immunological cellular response (Th1 cytokines). We found that the expression levels of IL-12, IL-18 and IL-21 were significantly diminished in malnourished children compared to well-nourished children and were coincident with lower plasmatic levels of IL-2 and IFN-γ (Th1 cytokines). In this study, we show for the first time that the gene expression and intracellular production of cytokines responsible for Th1 cell differentiation (IL-12, IL-18 and IL-21) are diminished in malnourished children. As expected, this finding was related to lower plasmatic levels of IL-2 and IFN-γ. The decreased expression of Th1 cytokines observed in this study may contribute to the deterioration of the immunological Type 1 (cellular) response. We hypothesize that the decreased production of IL-12, IL-18 and IL-21 in malnourished children contributes to their inability to eradicate infections.

## 1. Introduction

Malnutrition is either directly or indirectly responsible for 54% of the 10.8 million deaths per year in children under five in developing countries [1]. A diet consistently low in protein but with varying amounts of carbohydrates results in cells that lack the nutrients essential for normal metabolic functions. Moreover, malnutrition is a common cause of secondary immune deficiency and has been linked to an increased susceptibility to infection in humans [2].

It is well-known that malnutrition affects T-cell-mediated immune responses. Diminished T-cell immunological responses are generally associated with increased susceptibility to infections. This susceptibility is likely related to alterations in the production or expression of specific cytokines [3,4]. Cytokines are a large group of glycoproteins that can modulate the functional activity of individual cells under both physiological and pathological conditions [4]. These polypeptides are synthesized in response to microorganisms and other antigens, and they mediate and regulate the innate and adaptive immunological responses and act as inflammatory mediators or modulatory molecules during an infectious process. The production of cytokines is transitory and is usually limited to the duration of the stimulus [5]. 

Cytokines can be used to characterize T-cell responses as either Type 1 (Th1), associated with cell-mediated immunity, or Type 2 (Th2), associated with humoral immunity; this characterization provides a basis for understanding how T cells contribute to resistance or susceptibility to specific infections [6]. Functional differences between the Th subsets can largely be explained by the activities of the subset-specific cytokines produced. Specifically, Th1 cells are classified as cells that secrete IL-2 and IFN-γ, whereas Th2 cells are classified as cells that secrete IL-4 and IL-6. Additionally, IL-12, IL-18 and IL-21 are the cytokines responsible for the differentiation of Th1 cells. Th0 cells, which secrete cytokines typical of both Th1 and Th2 cells, can differentiate partially towards the Th1 and Th2 phenotypes. It has been proposed that Th0 cells are an intermediate cell type between precursor T cells and Th1 and Th2 cells [7]. It is well-known that the immunological cellular response is impaired in malnourished children, but this study is the first to examine the mechanisms involved in Th1 cell differentiation in these children in detail.

The aim of this study was to evaluate the expression levels of the cytokines involved in Th1 cell differentiation (IL-12, IL-18 and IL-21) by PCR and flow cytometry and to correlate this with the plasmatic levels of two Th1 cytokines (IL-2 and IFN-γ) in malnourished and well-nourished children hospitalized for bacterial infection. Additionally, we assessed the plasmatic levels of IL-12.

## 2. Materials and Methods

Heparinized peripheral blood samples were obtained from the Pediatric Hospital in Iztapalapa, Mexico and from the General Hospital of Mexico, Secretaria de Salud, Mexico from between December 2010 to March 2012. Informed consent was obtained from the parents of each child participating in this study. This study was assessed and approved by the Medical Ethics Committee of the General Direction of Medical Services of the Mexico City Government (Mexico) and by the Medical Ethics Committee of the Hospital General of Mexico.

### 2.1. Subjects

The samples of all the children included in the study were taken at the admission time to the hospital. All the children included in the study presented serious bacterial infections and required hospitalization. Bacterial infections in all cases were diagnosed rigorously based on clinical data and laboratory routine tests. The children with respiratory bacterial infections presented fever, cough and varying degrees of respiratory failure. The children with gastrointestinal bacterial infections presented diarrhea, fever and different degrees of dehydration. Bacterial agents were not determined in the children of the present study.

Four groups of children were studied:

***Group I: WN-I***
  This group included well-nourished children who were hospitalized because of a respiratory or gastrointestinal bacterial infection. All of the children had appropriate weight/height ratios for their ages.
***Group II: MN-1***
  This group included children who were hospitalized because of a respiratory or gastrointestinal bacterial infection. All of the children in this group had weight/height deficits of >10% and <25%.
***Group III: MN-2***
  This group included children who were hospitalized because of a respiratory or gastrointestinal bacterial infection and who had weight/height deficits of >25% and <40%.
***Group IV: MN-3***
  This group included children who were hospitalized because of a respiratory or gastrointestinal bacterial infection and who had weight/height deficits of >40%.

Weight/height deficits were determined according to the established values for Mexican children [8].

### 2.2. Blood Samples

Venous blood samples collected from each group of children were centrifuged for 10 min at 300× *g*. Plasma was obtained from a number of the samples, and aliquots of plasma were stored at −70 °C until being subjected analysis. The other samples were centrifuged as described above, and the resulting pellet was then processed to isolate mononuclear cells. It was not possible to use all the samples that were collected for all three analyses because the volumes of some of the samples were very low.

### 2.3. Gene Expression Analysis

Mononuclear cells in the pellet were separated using Linfograd density gradient centrifugation (Microlab, Mexico) for 20 min at 300× *g*.

The mononuclear cells were then lysed in a buffer containing guanidinium isothiocyanate, and the total RNA was isolated using an RNeasy kit (Qiagen, City, MD, USA). The isolated RNA was quantified using a Genesys™ 10 Series (ThermoSpectronic, Rochester, NY, USA) spectrophotometer, and 5 μg of RNA was separated on a 1.0% agarose gel containing ethidium bromide in a 3-(*N*-morpholino) propanesulfonic acid buffer. Both the running buffer and the gel contained 0.2 M formaldehyde. RNA samples were treated with amplification grade DNase I (Invitrogen, Carlsbad, CA, USA) before reverse transcription to remove trace amounts of DNA contamination. All of the RNA samples were stored at −80 °C in an RNA elution solution until further use.

RNA isolated from the samples collected from specific children (0.5 μg each sample) was used for reverse transcription primed with oligo-d(t) primers in 20-μL reaction volumes using Superscript III reverse transcriptase (Life Technologies, Rockville, MD, USA). The reactions were performed in an Eppendorf Mastercycler thermocycler (Eppendorf Scientific, Inc., Westbury, NY, USA). The amplified cDNA was quantified on a spectrophotometer at 260 nm.

Real-time PCR reactions were performed using the Human Universal ProbeLibrary (Roche Diagnostics GmbH, Mannheim, Germany). Specific oligonucleotide primers were originally generated using the online assay design software (ProbeFinder; http://www.universalprobelibrary.com [9]); the primer sequence for each gene is shown in Table 1. The cytokine gene expression levels were determined using real-time PCR. 

**Table 1:** Detailed Primers and Conditions Used for Real-Time PCR Assays.

Gene product	Primer name	Primer sequence	Annealing temp (°C)	Product size (bp)
IL-12A	IL-12-R	CACTCCCAAAACCTGCTGAG	60	88
	IL-12-F	TCTCTTCAGAAGTGCAAGGGTA		
IL-18	IL-18-R	CAACAAACTATTTGTCGCAGGA	60	73
	IL-18-F	TGCCACAAAGTTGATGCAAT		
IL-21	IL-21-R	AGGAAACCACCTTCCACAAA	60	68
	IL-21-F	GAATCACATGAAGGGCATGTT		
β-Actin	β-Actin-R	TCTACAACGAGCTGCGAAT	60	115
	β-Actin-F	CAATTTCCCTCTCGGCCTCG		

The 20-μL reaction mixture contained 1X LightCycler TaqMan Master reaction mixture (Roche Diagnostics, Mannheim, Germany), 200 nM of each primer, 100 nM of Universal ProbeLibrary probe, 0.5 U Light-Cycler Uracil DNA Glycosylase, and 2 μL of standard DNA in appropriate dilutions. Thermal cycling was initiated with 1 cycle at 95 °C for 60 s to denature the template and to perform a “hot start”, followed by 40 three-segment cycles to amplify the specific PCR product (95 °C for 10 s, the primer-specific annealing temperature for 30 s and product elongation at 72 °C for 18 s) and was terminated with 1 cycle at 40 °C for 30 s. The amplification was performed in borosilicate glass capillaries (Roche Diagnostics). The real-time PCR assay included serial log dilutions of the cDNA to generate a standard curve for each gene. The mean crossing threshold (Ct) of each gene was normalized to the mean Ct of the housekeeping gene β-actin. The data were analyzed using LightCycler^®^ Software version 4.0 [10].

### 2.4. Intracellular Cytokine Analysis

We used antibodies commercially conjugated to fluorescein isothiocyanate (FITC), phycoerythrin (PE), peridin-chlorophyll protein (PerCP) and allophycocyanin (APC) dyes (Becton Dickinson Immunocytometry System, San Jose, CA) in the following combinations: (1) isotype control; (2) CD14 FITC/IL-12 PE/CD45 PerCP; (3) CD3 FITC/IL-6 PE/CD45 PerCP; (4) CD4 FITC/IL-21 PE/CD45 PerCP; (5) IL-18 FITC/CD4 PE/CD45 PerCP/CD8 APC. 

Specific staining of the respective cell surface molecules was performed with anti-human CD45, CD4 and CD14. One hundred microliters of whole blood was incubated with 20 μL of a fluorescence-conjugated antibody for 30 min at room temperature in the dark. Nonspecific mouse immunoglobulin G1 antibodies were used as controls to establish background fluorescence. After incubation, the cells were washed with phosphate-buffered saline containing 1% bovine serum albumin.

Each sample that was analyzed was treated with fluorescence-activated cell sorter lysing solution. After further incubation, the samples were centrifuged and treated with fluorescence-activated cell sorter permeabilizing solution (Becton Dickinson, San Jose, CA, USA) for 10 min at room temperature. The samples were washed with 1% bovine serum albumin in phosphate-buffered saline and incubated with fluorescence-labeled anti-cytokine antibodies for 30 min. After incubation, the cells were washed and fixed in 1% paraformaldehyde prior to analysis with a FACSCalibur flow cytometer. A minimum of 10,000 events that fell within a cell-gate was acquired and analyzed using CELL Quest software (Becton Dickinson). 

### 2.5. Cytokine Plasmatic Analysis

For some children, the IL-2, IFN-γ, and IL-12 plasma levels were determined by ELISA (Human IL-2, IFN-γ and IL-12 [p70] ELISA Development Kits PeproTech), according to the manufacturer’s instructions. Briefly, the microtiter plates were coated with purified monoclonal antibodies against either human IL-2, IFN-γ or IL-12. The plates were then incubated overnight at room temperature. The liquid was aspirated from the wells, and the plates were washed 4 times using 300 μL of wash buffer per well. Three hundred microliters of blocking buffer were added to each well, followed by 1-hour incubation at room temperature. The wells were washed, and 100 μL of standard or sample was added to each well in duplicate. The plates were incubated at room temperature for 2 h. The wells were washed again and were then incubated with 100 μL of the detection antibody per well. The plates were incubated at room temperature for 2 h, and 100 μL per well of Avidin Peroxidase was then added and incubated for 30 min at room temperature. Finally, once the wells were washed and 100 μL ABTS liquid substrate (Sigma) was added to each well, the plates were incubated at room temperature to allow color development. The color development was monitored with an ELISA plate reader at 405 nm.

### 2.6. Statistical Analysis

In all cases, statistically significant differences between groups were established using the statistical Mann-Whitney U-test. *P* values ≤0.05 were considered to indicate significant differences between the compared groups.

## 3. Results

It was not possible to use all the samples that were collected for all three analyses because the volumes of some of the samples were very low.

### 3.1. Gene Expression Levels

The expression levels of the cytokines were compared among the WN-I group (11 girls and 5 boys, mean age 24.2 months, mean weight 12.1 kg, mean height 83.6, weight deficit <10%); the MN-1 group (5 girls and 5 boys, mean age 20.4 months, mean weight 9.2 kg, mean height 77.7, weight deficit range 13%–20%); the MN-2 group (3 girls and 4 boys, mean age 21.3 months, mean weight 8.4 kg, mean height 75.7, weight deficit range 25%–28%); and the MN-3 group (5 girls and 3 boys, mean age 20.4 months, mean weight 5.7 kg, mean height 69, weight deficit range 41%–68%). In all groups, the children were hospitalized because of a respiratory or gastrointestinal bacterial infection, as indicated above. 

The expression results were standardized with respect to the reference gene β-actin, and the relative expression levels of IL-12, IL-18 and IL-21 for children in groups WN-I, MN-1, MN-2 and MN-3 were analyzed. The data are expressed as the comparison between a WN-I child and an MN child with a similar type of infection and of a similar age. 

**Figure 1:**
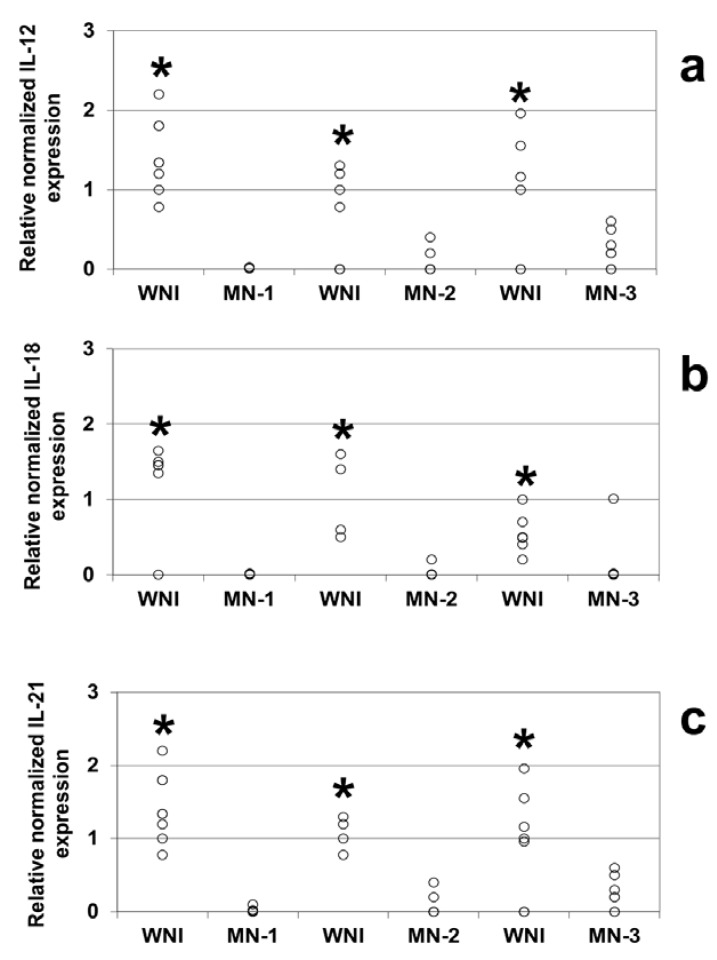
Gene expression levels from well-nourished and malnourished children, IL-12 (**a**), IL-18 (**b**), and IL-21 (**c**) levels were obtained by Real Time PCR. Mononuclear cells were obtained from peripheral blood samples of WNI, MN-1, MN-2 and MN-3. Determinations were made in triplicate, and the points in the graph represent their average. Significant difference: In all cases * WNI *vs.* MN-1, MN-2 and MN-3, *p* < 0.05. IL-12: BNI *vs.* DN1 *n* = 6; BNI *vs.* DN2 *n* = 5; BNI *vs.* DN3 *n* = 6. IL-18: BNI *vs.* DN1 *n* = 6; BNI *vs.* DN2 *n* = 4; BNI *vs.* DN3 *n* = 7. IL-21: BNI *vs.* DN1 *n* = 7: BNI *vs.* DN2 *n* = 4; BNI *vs.* DN3 *n* = 7.

Comparison of the relative expression levels of different genes revealed that IL-12 (Figure 1a), IL-18 (Figure 1b) and IL-21 (Figure 1c) were expressed at lower levels in all of the malnourished groups studied compared to the WN-I children (*p* < 0.05). 

### 3.2. Intracellular Cytokine Analysis

Intracellular cytokine expression was examined in 12 WN-I children (6 girls and 6 boys, mean age 27.3 months, mean weight 12.9 kg, mean height 85.8, weight deficit <10%); 11 MN-1 children (7 girls and 4 boys, mean age 17.3 months, mean weight 8.7 kg, mean height 74.8, weight deficit range 11%–24%); 11 MN-2 children (5 girls and 6 boys, mean age 19.6 months, mean weight 7.7 kg, mean height 77, weight deficit range 25%–38%) and 6 MN-3 children (4 girls and 2 boys, mean age 12.2 months, mean weight 5.2 kg, mean height 57.6, weight deficit range 41%–51%).

**Figure 2:**
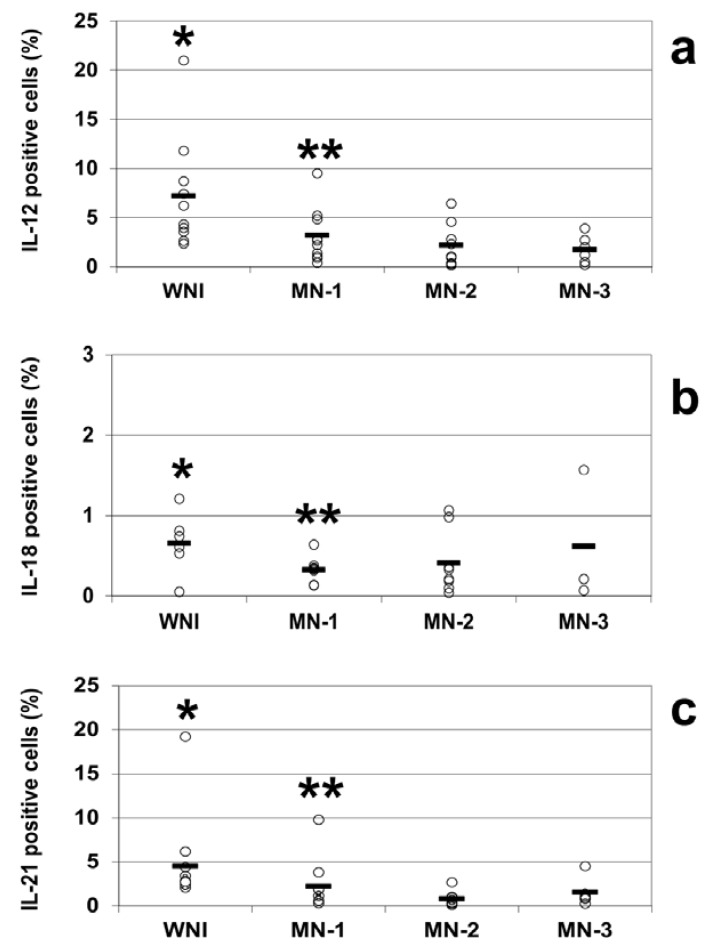
Percentages of positive cells from well-nourished and malnourished children, IL-12 (**a**), IL-18 (**b**), and IL-21 (**c**) were analyzed by flow cytometry in WNI, MN-1, MN-2 and MN-3. Cells were stained and analyzed as described in the Methods section. Data are based upon flow cytometric analysis of 10,000 events. Results are expressed as percentage of positive cells and are showed individual data and the mean represented by the bar. Significant difference: IL-12 and IL-21. * WNI *vs.* MN-1, MN-2 and MN-3; IL-18 WN-I *vs.* MN-2 and MN-3, ** MN-1 *vs.* MN-3; *p* < 0.05.

The intracellular cytokine analysis indicated decreases in the percentages of IL-12-positive cells in all of the groups of malnourished infected children compared to WN-I children. The percentage of IL-12-positive cells in WN-I children was 7.2% ± 1.5 (mean ± SE); for the MN-1 group, it was 3.2% ± 0.8, and for the MN-2 group, it was 2.2% ± 0.7. The lowest percentage of IL-12-positive cells was observed in the MN-3 group, with 1.7% ± 0.6 IL-12-positive cells. All of the groups of malnourished children showed statistically significant reductions in the percentage of IL-12-positive cells compared to the WN-I children (*p* < 0.05; Figure 2a). 

The percentages of IL-21-positive cells were diminished in all of the groups of malnourished children compared to well-nourished infected children, similar to the percentages observed for IL-12. In the WN-I group, the percentage of IL-21 cells was 4.5% ± 1.4; for the MN-1 group, it was 2.3% ± 0.9, for the MN-2 group, it was 0.8 ± 0.3 and for the MN-3 group, it was 1.5% ± 0.6. All of the groups of malnourished children showed statistically significant reductions in the percentage of IL-21-positive cells compared to the WN-I children (*p* < 0.05; Figure 2c).

The percentages of IL-18-positive cells in well-nourished and malnourished children were notably small; however, there was again a trend towards lower percentages in the malnourished children: 0.7% ± 0.2 in the WNI group; 0.3% ± 0.1 in the MN-1 group; 0.4% ± 0.1 in the MN-2 group; and 0.6% ± 0.5 in the MN-3 group. There were statistically differences between the WN-I and the MN-2 and MN-3 groups and between the MN-1 and MN-3 groups (*p* < 0.05 in all comparisons; Figure 2b).

### 3.3. Plasmatic Cytokine Levels

The analysis of the plasmatic concentration of cytokines was performed in 10 children in the WN-I group (3 girls and 7 boys, mean age 28.7 months, mean weight 14.2 kg, mean height 99.9, weight deficit <10%); 7 children in the MN-1 group (3 girls and 4 boys, mean age 30.3 months, mean weight 10.9 kg, mean height 88.3, weight deficit range 13%–20%); 10 children in the MN-2 group (2 girls and 8 boys, mean age 13.5 months, mean weight 6.9 kg, mean height 68.5, weight deficit range 25%–37%) and 7 children in the MN-3 group (3 girls and 4 boys, mean age 18.6 months, mean weight 6.2 kg, mean height 72, weight deficit range 41%–47%). In all groups, the children were hospitalized because of a respiratory or gastrointestinal bacterial infection, as we mentioned previously.

**Figure 3:**
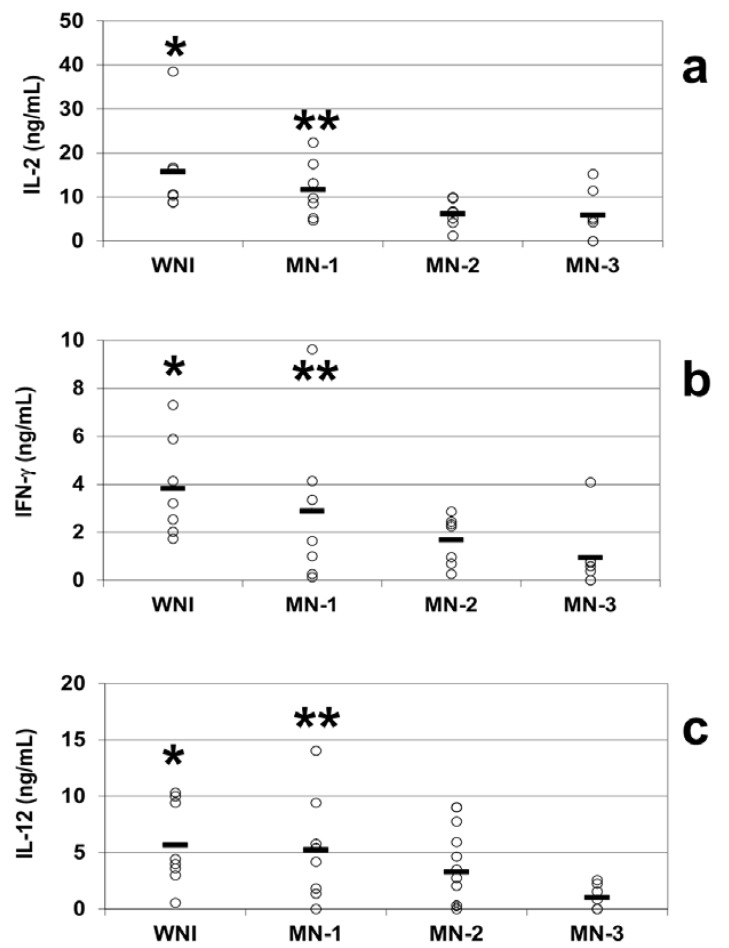
Plasma cytokine levels from well-nourished and malnourished children. Circulating IL-2 (**a**), IFN-γ (**b**) and IL-12 (**c**) levels were obtained by ELISA. The plasma was obtained from peripheral blood samples of WNI, MN-1, MN-2 and MN-3. The data are presented as the mean ± standard error. (**a**) Plasma IL-2 levels. Significant difference: * WNI *vs.* MN-2 and MN-3; ** MN-1 *vs.* MN-3; *p* < 0.05; (**b**) Plasma IFN-γ levels. Significant differences: * WNI *vs.* MN-2 and MN-3, ** MN-1 *vs.* MN-3, *p* < 0.05; (**c**) Plasma IL-12 levels. Significant difference: * WNI *vs.* MN-2 and MN-3; ** MN-1 *vs.* MN-3; *p* < 0.05.

We observed decreases in the mean plasmatic levels of IL-2 in all of the groups of malnourished infected children compared to the well-nourished infected children. The mean plasma concentrations of IL-2 were 15.7 ± 4.7 ng/mL in the WN-I children, 11.6 ± 2.5 ng/mL in the MN-1 group, 6.1 ± 1.3 ng/mL in the MN-2 group and 5.8 ± 2.1 ng/mL in the MN-3 group. The MN-2 and MN-3 groups were statistically significantly different from the WN-I group (*p* < 0.05; Figure 3a).

The plasmatic IFN-γ levels were 3.8 ± 0.8 ng/mL in the WN-I children, while the malnourished groups exhibited mean levels of 2.9 ± 1.3 ng/mL in the MN-1 group, 1.7 ± 0.4 ng/mL in the MN-2 group and 0.9 ± 0.5 ng/mL in the MN-3 group. The most malnourished children exhibited the lowest levels of plasmatic IFN-γ. There were significant decreases (*p* < 0.05) in the levels of IFN-γ in the plasma of the children in the MN-2 and MN-3 groups compared to the WN-I group (Figure 3b).

**Figure 4:**
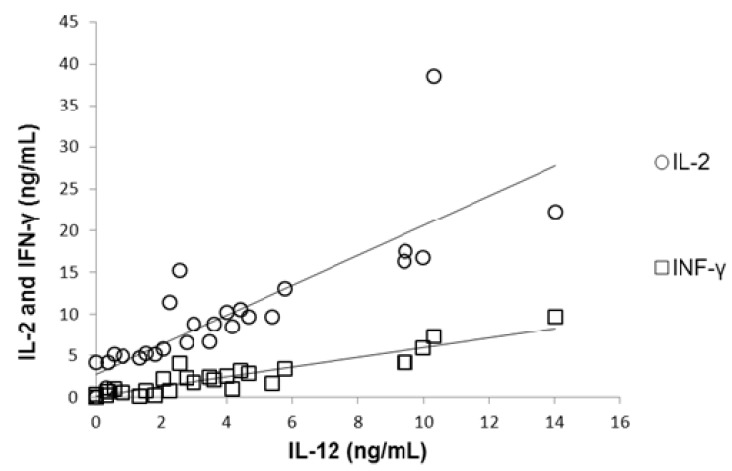
Correlation between plasmatic concentration of IL-12 and IL-2 (*r* = 0.92; *p* < 0.01) and IFN-γ (*r* = 0.89; *p* < 0.01). Malnourished children showed the lowest concentration of IL-12 and this was associated with diminished concentrations of IL-2 and IFN-γ.

The plasma levels of IL-12 were diminished in direct relation to the severity of malnutrition in malnourished children compared to the children in the WN-I group, who had IL-12 plasma levels of 5.7 ± 1.3 ng/mL. Similar to the pattern observed for IFN-γ, the IL-12 circulating mean levels observed in malnourished groups were 5.3 ± 1.6 ng/mL in the MN-1 group and 3.3 ± 0.9 ng/mL in the MN-2 group. The lowest IL-12 plasma levels (1.03 ± 0.4 ng/mL) were observed in the most severely malnourished children. The IL-12 plasma levels in the MN-2 and MN-3 groups differed statistically significantly from those in the WN-I children (*p* < 0.05; Figure 3c). Correlation between plasmatic concentration of IL-12 and IL-2 and IFN-γ was established (Figure 4), results show that a diminished concentration of IL-12 (as in MN children) was correlated with diminished values of IL-2 (*r* = 0.92; *p* < 0.01) and IFN-γ (*r* = 0.89; *p* < 0.01). 

## 4. Discussion

The relationship between nutritional status and the immune system has been a topic of study for decades. Multiple abnormalities in the immune response in malnourished children have been described and could account for the increased severity and frequency of infections observed in these children [2]. It is well known that malnutrition is a major cause of secondary immune deficiency in the world [11] and that malnutrition in children is associated with an increased risk of infection and death [12]. 

We have previously linked impaired lymphocyte function in malnourished children to a down-regulation of Type 1 cytokines such as IL-2 and IFN-γ and a marked up-regulation of Type 2 cytokines such as IL-4 and IL-10 [3,4]. In this study, we extended these findings by examining the expression of IL-12, IL-18, and IL-21 at the gene expression and protein levels and the presence of IL-12 in the plasma—all cytokines involved in Th1 differentiation—and correlating these expression levels with the circulating levels of two Type 1 cytokines, IL-2 and IFN-γ. The generation of a protective T-cell response against infectious agents is a complex process in which cytokines provide signals directing the development of adaptive immunity [13].

Cytokines play a critical role in Th cell polarization. It is well known that IL-12, IL-18, and IL-21 [14] control Th1 cell differentiation. The differentiation of Th1 cells is critical for the immunological competence of an organism because these cells are responsible for the production of IFN-γ, IL,-2 and TNF-α, which mediate delayed-type hypersensitivity responses and macrophage activation [15]. In this study, we observed that the gene and protein expression levels of IL-12, IL-18, and IL-21 and the plasmatic level of IL-12 were diminished in malnourished children. Possibly as a consequence of this, we observed lower plasmatic concentrations of IFN-γ and IL-2. Our results demonstrate that T lymphocytes from malnourished children have an impaired capacity for RNA and protein synthesis of cytokines, which results in a diminished capacity to secrete cytokines. These findings are relevant because they contribute to our understanding of the altered immunological status of malnourished children.

In agreement with our data, previous studies indicate that malnutrition decreases T-cell function, cytokine production, and the capacity of lymphocytes to respond appropriately to cytokines (Rodríguez *et al.*, 2005 [3]; Hoffman-Goetz, 1988 [16]; Pelletier *et al.*, 1993 [17]). Malnourished children have been shown to have altered capacities to produce several cytokines (*i.e.*, IL-2, IL-4, IL-6, IL-10, *etc.*). In relation to this, González *et al.* (1997) [18] observed that lymphocytes obtained from malnourished children were unable to secrete normal quantities of cytokines or to achieve adequate immunologic function and proposed that the altered physiology of lymphocytes may be the predominant cause of the immune impairment observed in malnourished children.

IL-12, IL-18, and IL-21 cooperate to stimulate the differentiation of naïve Th cells into Th1 cells, which is fundamental to the immunocompetence of an individual. However, these cytokines have multiple functions, and their decreased expression in malnourished children may affect other immunological functions. 

IL-12 is also known as a natural killer (NK) cell stimulatory factor and a cytotoxic lymphocyte maturation factor and is a pleiotropic proinflammatory cytokine that is produced primarily by antigen-presenting cells (APCs) [19]. IL-12 can induce the production of Th1 cytokines and can suppress the production of Th2 cytokines [20]. It has multiple effects on T lymphocytes and can stimulate cytotoxicity, proliferation, cytokine production and the development of Th1 subsets [21]. IL-12 is not only an important proinflammatory cytokine, inducing the production of IFN-γ and the subsequent activation of phagocytic cells; it also plays a major role in regulating the migration and proper positioning of effector cells. IL-12 facilitates Th1 responses by stimulating the differentiation of naïve Th cells into Th1 cells and by serving as a required co-stimulus for maximum IFN-γ secretion by antigen-activated Th1 cells [22]. A preceding study reported reduced IL-12 production by dendritic cells (DCs) in severely malnourished children. The impaired production of IL-12 inhibits T cell proliferation and effector functions [23], which have been extensively demonstrated in malnourished children. In this study, we found that the gene expression, intracellular production and plasmatic concentration of IL-12 were diminished in children in the MN-I group; pathologic changes which may globally affect the immunological function of malnourished children.

It has been shown that IL-12 and IL-18, which are synthesized by activated macrophages, synergistically stimulate IFN-γ production by T and NK cells [24]. However, in other studies, only IL-12 was strongly correlated with IFN-γ production [25]. The data obtained in this study suggest that the decreased production of IL-12 and IL-18 in the MN-2 and MN-3 groups may be related to the reduced production of IFN-γ observed in the same groups of children. 

IL-18 both serves as a cofactor for IL-12-induced Th1 development and enhances IFN-γ production from effector Th1 cells. IL-18 is a member of the IL-1 family and is primarily produced by T lymphocytes, macrophages and DCs. IL-18 also modulates Th1 development. Although IL-18 alone cannot induce Th1 cell differentiation, it strongly augments IL-12-dependent Th1-cell development and effector functions, probably by upregulating IL-12Rβ2 chain expression on T cells and AP-1 (c-fos/c-jun)-dependent trans-activation of the IFN-γ promoter [14]. In the absence of IL-12, IL-18-mediated effects on T cells may extend beyond Th1 differentiation to include Th2 cytokine production [26]. 

Although IL-18 or IL-21 alone induced modest expression of the IFN-γ gene, a combination of IL-21 and IL-18 efficiently up-regulated IFN-γ production [27]. Additionally, IL-21 regulates the differentiation and function of effector CD4^+^ T helper cells; controls the activation, proliferation, and survival of B cells; and enhances the cytotoxic activity of CD8^+^ T cells and NK cells. IL-21 also inhibits the differentiation of inducible regulatory T cells (Tregs) and makes effector CD4^+^ T cells resistant to Treg-mediated immunosuppression [28].

In relation to IL-2, our data revealed decreased plasmatic levels of IL-2 in children in the MN-2 and MN-3 groups. IL-2 is well known for its role in T-cell activation and expansion [29]. IL-2 is critical for the development and peripheral expansion of CD4^+^CD25^+^ regulatory T cells, which promote self-tolerance by suppressing T cell responses *in vivo* [30]. In accordance with our observations, previous data clearly demonstrate a decrease in both the intracellular production [11] and the gene expression [14] levels of IL-2 in malnourished children. In this study, we show that the secretion of IL-2 is also diminished in malnourished children.

In this report, we demonstrated that circulating levels of IFN-γ are also diminished in the plasma of children in the MN-2 and MN3 groups. IFN-γ is an important proinflammatory cytokine produced during Type 1 T-lymphocyte helper responses, which increases the expression of MHC class I and class II molecules. The IFN-γ produced by T cells may also activate mononuclear phagocytes (monocytes and DCs). Macrophages are activated by a variety of stimuli, including IFN-γ, which induces the differentiation and activation of monocyte-macrophages and enhances their microbicidal activity [31]. Additionally, data from our previous studies revealed significant decreases in the relative expression of the IFN-γ gene [4] and in IFN-γ production by T cells from malnourished children [3]. 

This study demonstrates that the alterations observed in the Th1 responses in malnourished children are the consequence of impaired expression of cytokines at the gene and protein levels and diminished plasmatic levels of IL-12, IL-18, and IL-21. The data also indicate that immunological function is more impaired in children in the MN-2 and MN-3 groups.

## 5. Conclusion

The results of this investigation demonstrate for the first time that decreases in the gene and protein expression levels of cytokines involved in Th1 cell differentiation (IL-12, IL-18, IL-21) are related to diminished production of IL-2 and IFN-γ (Th1 cytokines) in malnourished children. This study helps to elucidate the mechanisms involved in the deterioration of the immunological Type 1 (cellular) response in malnourished children. 
